# Blood Serum From Obese Women Raises ROS Production by Neural Stem Cells

**DOI:** 10.1002/dneu.70021

**Published:** 2026-03-17

**Authors:** Phelipe Elias da Silva, Natássia Caroline Resende Corrêa, Natália Ferreira Silva, Carlos Ueira‐Vieira, Hebreia Oliveira Almeida‐Souza, Mario Machado Martins, Tiara da Costa Silva, Renata Graciele Zanon

**Affiliations:** ^1^ Institute of Biomedical Sciences Federal University of Uberlandia Uberlandia Minas Gerais Brazil; ^2^ Institute of Biotechnology Federal University of Uberlandia Uberlandia Minas Gerais Brazil; ^3^ Institute of Biology State University of Campinas Campinas São Paulo Brazil

**Keywords:** cell differentiation, cell viability, embryonic stem cells, neural stem cells, obesity, oxidative damage

## Abstract

Maternal obesity has been associated with adverse pregnancy outcomes and altered fetal development, but the direct influence of circulating maternal factors on early human neural cells remains poorly understood. Neural stem cells (NSCs) provide a controlled system to examine how metabolic and inflammatory changes may affect early neurodevelopment. We differentiated human embryonic stem cells into NSCs and exposed them to 10% serum from non‐obese or obese women. Cell viability, oxidative stress, metabolic activity, proliferation, and neural marker expression were evaluated. Metabolomic profiling confirmed distinct serum signatures between donor groups, particularly involving lipid and redox‐related metabolites. Exposure to human serum, independent of donor phenotype, reduced viability, decreased Ki‐67 and PAX6 expression, increased Caspase‐3 and p53 labeling, and altered progenitor markers, indicating activation of stress pathways. Although overall responses to non‐obese and obese serum were similar, NSCs exposed to obese serum showed a sustained increase in ROS and a transient elevation in resazurin reduction at later time points. These differences were modest but statistically significant and may reflect altered metabolic and redox handling. Together, the findings show that serum exposure imposes considerable stress on NSCs in vitro and that obesity‐related factors may subtly amplify oxidative responses. The study also underscores the limitations of this artificial model and highlights the need for systems that more closely approximate physiological conditions during neurodevelopment.

## 1 Introduction

Obesity, a multifactorial condition with rapidly rising global prevalence, is projected to affect a large proportion of adults by 2030 (Ng et al. 2014; Seravalle and Grassi 2017; Lin and Li 2021). During pregnancy, maternal obesity increases the risk of complications such as gestational diabetes and hypertension and is linked to long‐term metabolic and neurological vulnerabilities in offspring (Macinnis et al. 2016; Villamor and Cnattingius 2006; Wu et al. 2023). Experimental studies also show that maternal obesity can alter the structural and transcriptional development of the fetal hypothalamus, emphasizing the sensitivity of early neurodevelopment to maternal metabolic status (Dearden et al. 2020). This is particularly relevant during neural tube formation, a stage that relies on precise maternal‐embryonic exchanges of nutrients and signaling molecules (Anderson et al. 2005; Singh and Munakomi 2023).

Evidence from animal models indicates that maternal obesity interferes with neurodevelopmental pathways, including the maturation of neural stem cells (NSCs). It can disrupt cytokine signaling networks such as IL‐6, which regulates neuroepithelial self‐renewal (Davis and Mire 2021), stimulate abnormal proliferation and differentiation of early neural precursors (Chang et al. 2008), and reduce NSC pools in offspring, accelerating premature neuronal differentiation (Urbonaite et al. 2022). Although informative, these findings cannot be directly translated to humans due to species‐specific developmental differences (P. Wang et al. 2016; Zeiss 2021; Ma et al. 2022; Cottam et al. 2024).

To address this gap, human pluripotent stem cells (PSCs), including embryonic stem cells (ESCs), and induced pluripotent stem cells (iPSCs), have become invaluable tools for modeling human neural development (Crook et al. 2015; Hayashi et al. 2019). Their ability to generate multiple neural lineages has accelerated the transition from two‐dimensional differentiation systems to three‐dimensional cerebral organoids that mimic aspects of early human brain organization (Lancaster and Knoblich 2014; Velasco et al. 2019; Weatherbee et al. 2021).

Cerebral organoids enable the systematic investigation of genetic mutations and patient‐specific disease phenotypes using induced PSCs‐derived models (Nishimura et al. 2024). Despite limitations such as the lack of a functional vascular network, which restricts their ability to fully reproduce human brain architecture (Y. Zhao et al. 2025), organoids remain powerful systems for elucidating mechanisms driving neurodevelopmental, neurodegenerative, and infectious disorders (Eichmüller and Knoblich 2022) and for examining how maternal factors, including those associated with obesity, may influence early NSC behavior (Sarieva and Mayer 2021).

Neuroectodermal regions within these models form neural rosettes, structures mimicking the early neural tube, containing multipotent NSCs capable of generating neurons, astrocytes, and oligodendrocytes (Xiang et al. 2021; Whye et al. 2023; Miotto et al. 2023). These properties make NSCs a relevant in vitro system for assessing developmental neurotoxicity (Al‐Rubai et al. 2017).

Accumulating evidence indicates that NSCs are closely integrated with the developing cerebral vasculature, challenging the view that these cells are spatially segregated from vascular influences. In adult neurogenic niches, seminal studies demonstrated that NSCs within the subventricular zone are intimately associated with capillaries, forming specialized vascular niches in which endothelial‐derived cues regulate stem cell quiescence, activation, and maintenance (Tavazoie et al. 2008; Shen et al. 2008; Bond et al. 2015; Karakatsani et al. 2019). During embryonic brain development, these interactions are progressively established as the neuroepithelium undergoes vascularization by the perineural plexus, and angiogenic signaling, relief of hypoxia, and endothelial‐derived factors critically modulate NSC proliferation, lineage commitment, and metabolic state (Hogan et al. 2004; Javaherian and Kriegstein 2009; Paridaen and Huttner 2014; Paredes et al. 2018; Tata and Ruhrberg 2018). In this developmental context, the initial structural and functional immaturity of the blood–brain barrier (BBB) permits maternal serum‐derived molecules to access the embryonic neural microenvironment, thereby increasing NSC susceptibility to systemic metabolic and inflammatory perturbations during critical windows of neurogenesis, with potential long‐term consequences for neurovascular and neuroimmune maturation in the offspring (Saunders et al. 2012; Haddad‐Tóvolli et al. 2017; Q. Zhao et al. 2022).

Complementary in vitro models have also been used to simulate obesogenic environments and dissect the actions of circulating cytokines and metabolic mediators (Ahluwalia et al. 2018; T. Wang and He 2018; Qiao et al. 2020; Ghanemi et al. 2022).

This collective evidence highlights the need for human‐relevant systems that can directly test how maternal metabolic signals influence early neural development. In this context, the present study generated NSCs from hESCs and exposed them to serum from non‐obese and obese women to evaluate effects on viability, oxidative stress, proliferation, and neural marker expression. Although this approach does not model physiological barriers to maternal–fetal exchange, it provides a controlled stress paradigm for identifying cellular vulnerabilities and differential responses to circulating factors. These insights may contribute to hypotheses regarding how maternal metabolic states affect early neurodevelopment and inform future work in maternal–fetal health.

## 2 Materials and Methods

### 2.1 Cultivation of ESCs and Induction of NSC Differentiation

Human ESCs from the BR1 lineage were derived from surplus embryos obtained through voluntary donation following in vitro fertilization procedures, in accordance with Brazilian legislation (Biosafety Law No. 11,105, March 25, 2005). The use of this material was authorized upon signed informed consent from the donors and approved by the Ethics Committee of the Institute of Biosciences, University of São Paulo (Fraga et al. 2011).

**FIGURE 1:**
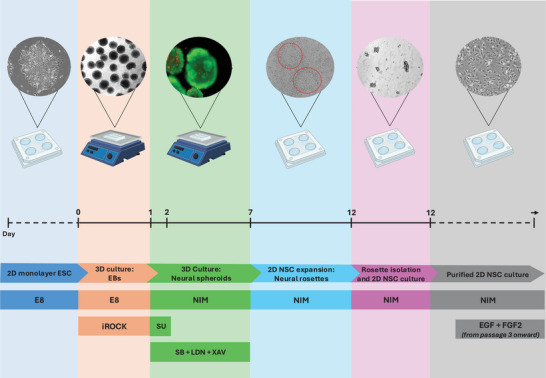
Timeline and schematic overview of neural induction and NSC derivation from human ESCs. hESCs (BR1 lineage) were initially maintained as 2D monolayers in E8 medium. Neural induction was initiated by EB formation under low‐adhesion conditions with orbital shaking (100 rpm) for 24 h in E8 medium. After an additional six days of neural induction, with EBs cultured in NIM supplemented with specific signaling pathway inhibitors under orbital shaking (120 rpm), neural spheroids were formed and subsequently transferred to Geltrex‐coated plates for 2D expansion, leading to the emergence of neural rosettes. NSCs were isolated from rosettes after 5 days and expanded in adherent culture for purification. From the third passage onward, EGF and FGF2 were added to the NIM to maintain NSC multipotency. The timeline illustrates the main culture stages, medium changes, and key experimental transitions during neural differentiation.

BR1 were cultured in 6‐well plates (Greiner, 657160, Greiner Bio‐One, Frickenhausen, Germany) previously coated with Geltrex (Gibco, A1413302, Life Technologies, Burlington, Canada), and maintained in Essential 8 medium (Gibco, A1517101, Life Technologies) at 37°C in a humidified incubator until reaching 80%–90% confluency. Cells were passaged using Accutase (Invitrogen, 00‐4555‐56, Life Technologies, Burlington, Canada), and the apoptosis inhibitor iROCK (StemCell, Y‐27632, StemCell Technologies, Vancouver, Canada) was added to the E8 medium during dissociation. For neural differentiation (Figure 1), BR1 human ESC were dissociated and seeded onto low‐adhesion plates under orbital shaking for 24 h in E8 medium supplemented with iROCK to induce embryoid body (EB) formation. Subsequently, EBs were maintained in neural induction medium (NIM) (Table 1), supplemented with specific inhibitors (Figure S1), under orbital shaking for an additional six days.

**TABLE 1:** Composition for the preparation of 42.5 mL of NIM.

Trade name	Quantity used	Commercial specification
DMEM F12 medium	20 mL	Gibco, 11320033, Life Technologies, Burlington, Canada
Neurobasal medium	20 mL	Gibco, 10888022, Life Technologies, Burlington, Canada
N‐2 Supplement (100X),	400 µL	Gibco, 17502048, Life Technologies, Burlington, Canada
B‐27 Supplement (50X), minus vitamin A	800 µL	Gibco, 12587010, Life Technologies, Burlington, Canada
Glutamax I (100X)	400 µL	Gibco, 35050061, Life Technologies, Burlington, Canada
Pen Strep (100X)	400 µL	Gibco, 15240062, Life Technologies, Burlington, Canada
MEM Non‐Essential Amino Acids (100X)	400 µL	Gibco, 11140050, Life Technologies, Burlington, Canada
2‐Mercaptoethanol (1000X)	23,8 µL	Gibco, 21985023, Life Technologies, Burlington, Canada
l‐Ascorbic acid (200 mM)	40 µL	Sigma, A4403, Sigma‐Aldrich Brazil Ltda., São Paulo, Brazil

After the initial seven‐day induction period, neural spheroids were transferred to Geltrex‐coated plates for 2D expansion of NSCs. Neural rosettes containing NSCs became visible within 48 h under these culture conditions. After 5 days, NSCs were isolated from the rosettes and transferred to adherent culture for purification. From the third passage onward, epidermal growth factor (EGF) and fibroblast growth factor 2 (FGF2) were added to preserve multipotency. Differentiation efficiency was confirmed by real‐time PCR (RT‐qPCR) and immunocytochemistry, using markers such as *POU5F1* (*OCT4*), *NANOG*, *FABP7*, SOX1, *SOX2*, PAX6, NESTIN, and FOXG1.

### 2.2 RT‐qPCR

Total RNA was extracted from up to 1 × 10^6^ cells per experimental condition using TRIzol reagent (Invitrogen, 15596026, Life Technologies). RNA quantity was assessed by spectrophotometry (Nanodrop), and integrity was confirmed via 1% agarose gel electrophoresis. Reverse transcription was performed using the GoScript Reverse Transcription Mix, Oligo(dT) (Promega, A2790, Promega Corporation, Madison, USA). The resulting cDNA was amplified using gene‐specific primers (Table 2), previously validated for expression stability. RT‐qPCR reactions were carried out using the PrimeTime Gene Expression Master Mix (IDT, 1055772, Integrated DNA Technologies, Inc., Coralville, USA) on the CFX96 Real‐Time PCR Detection System (Bio‐Rad), with 25 ng of cDNA per reaction. Absolute gene expression levels were determined using a standard curve generated from serial dilutions of synthetic gBLOCK fragments (Integrated DNA Technologies, Inc.), amplified in triplicate under the same conditions as the experimental samples.

**TABLE 2:** Commercial qPCR assays and associated gene functions analyzed in this study. Primer and probe sequences are proprietary to the manufacturer.

Gene Name	qPCR assay ID (PrimeTime, IDT)	Gene function
*SOX2*	Hs.PT.58.237897.g	Maintenance of pluripotency (ESCs); Regulation of neurogenesis (NSCs) (Zhou et al. 2016)
*POU5F1 (OCT4)*	Hs.PT.58.14648152.g	Activation of pluripotency enhancers (L. Xiong et al. 2022)
*NANOG*	Hs.PT.58.21480849	Key regulatory factor for pluripotency and self‐renewal in ESCs (W. Zhang et al. 2016)
*NES (NESTIN)*	Hs.PT.58.1185097	Gene expression control in neural progenitor cells (Thomson et al. 2021)
*FABP7*	Hs.PT.58.40673612	Maintenance of neuroepithelial cells during cortical development, modulated by Pax6 (Arai et al. 2005)

### 2.3 Serum Collection and Treatment

Serum samples were obtained from 12 women of reproductive age, 6 classified as non‐obese and 6 as obese. Donors in the non‐obese group had a mean age of 34 years (±2.75 SD), and those in the OB group had a mean age of 35.17 years (±7.75 SD). Collections were performed in the morning following a minimum fasting period of 3 h. Non‐obese participants, with body mass index (BMI) ranging from 18.5 to 24.9 kg/m^2^, were healthy volunteers, including students and staff from the Federal University of Uberlândia (UFU). Obese participants, with BMI between 30 and 39.9 kg/m^2^, were recruited from the Endocrinology and Bariatric Surgery Outpatient Clinic of the UFU University Hospital. All obese participants were free from metabolic comorbidities or a family history of cancer. The study protocol was approved by the Institutional Research Ethics Committee (CAAE: 45449821.2.0000.5152). Women diagnosed with chronic inflammatory conditions, recent infections, neoplasms, or neurodegenerative diseases were excluded. For each participant, 12 mL of peripheral blood was collected, centrifuged at 3400 rpm for 15 min, and serum was aliquoted into microtubes and stored at −80°C.

NSCs were seeded in 96‐well Geltrex‐coated plates (Gibco) at a density of 7.5 × 10^3^ cells per well and allocated into three experimental groups: Control (CT), non‐obese (NO), and obese (OB). Before treatment, serum was filtered through a sterile syringe filter with a 0.22 µm pore size. Each serum sample was tested in triplicate (*n* = 6 per group). After 24 h of culture, the NIM was replaced, and 10% human serum was added to the appropriate wells, while the control group received only fresh NIM. For the resazurin reduction assay, reactive oxygen species (ROS) analysis, and propidium iodide (PI) staining, cells were exposed to serum for 24, 48, and 72 h without media replacement. Immunocytochemical analyses were performed following 72 h of treatment.

Human serum was applied at 10% to approximate systemic exposure to circulating maternal factors, a concentration frequently used in vitro to investigate serum‐driven cellular responses (Sylvester‐Armstrong et al. 2024; Ghebosu et al. 2021). All groups were subjected to the same serum concentration. Heat inactivation was not performed to preserve biologically active components, and background controls consisting of medium plus 10% serum without cells were included in fluorescence assays to account for matrix‐related signal interference.

### 2.4 Metabolomic Analysis of Serum

Metabolomics is an analytical approach that enables the comprehensive identification and quantification of low‐molecular‐weight metabolites (<1500 Da) in biofluids, contributing significantly to biomarker discovery and to the understanding of molecular mechanisms underlying diseases (Jacob et al. 2019). In this study, metabolomic profiling of serum samples from both experimental groups was performed by liquid chromatography coupled with mass spectrometry (LC‐MS), following metabolite extraction with 1000 µL of spectroscopic‐grade methanol added to 100 µL of serum, centrifugation (13,000 × *g*, 15 min), vacuum evaporation, and resuspension in 400 µL of methanol. Samples were filtered (0.22 µm) and analyzed on an Agilent Infinity 1260/Q‐TOF 6520 B system with electrospray ionization, using an Agilent Poroshell 120 column (2.1 × 50 mm, 2.7 µm) and mobile phases consisting of water with 0.1% formic acid and methanol, under a gradient from 2% to 98% of phase B. Acquisition parameters included a nebulizer pressure of 20 psi, drying gas flow at 8 L/min at 220°C, and a capillary voltage of 4.5 kV. Data were processed in MassHunter Qualitative Analysis (v.10.0) using the MFE tool and further analyzed in Mass Profiler Professional (v. B.13.1.1), applying filters of a minimum abundance of 5000 counts and a mass window of 15 ppm + 2 mDa. Compounds detected in 100% of the samples from at least one group were considered, and identification was performed based on the METLIN 2019 database.

### 2.5 Dual Calcein/PI Staining and Viability Assessment

Cell viability was assessed using dual fluorescent staining with Calcein, AM (3 µM; Invitrogen, C1430) for viable cells and PI (2.5 µM; Invitrogen, P1304MP) for non‐viable cells across different time points and experimental conditions. In addition, PI staining was analyzed separately to assess loss of membrane permeability. Stains were added directly to wells without washing, followed by a 30‐min incubation in the dark. Images were acquired using the EVOS M3000 microscope in brightfield and fluorescence (Texas Red: Ex 585 nm/Em 628 nm; GFP: Ex 470 nm/Em 525 nm) for simultaneous visualization. ImageJ (Schneider et al. 2012) was used for quantification via threshold adjustment, binary watershed segmentation, and particle analysis with predefined cell size limits. Viability was calculated as the percentage of viable cells relative to the total cell count (viable + non‐viable) per image.

### 2.6 Resazurin Reduction

Metabolic activity was evaluated through a resazurin reduction assay in cultures maintained in NIM medium supplemented with distinct serum samples. After washing each well in both experimental and control groups, resazurin solution (Sigma, 199303, Sigma‐Aldrich Brazil Ltda.) was added to achieve a final concentration of 0.3 µM. Wells containing only NIM were used to determine background fluorescence. As a negative control, 0.1% Triton X‐100 (Sigma, X100RS) was added to designated wells. Plates were incubated in the dark for 2 h, and fluorescence was measured using a VICTOR Nivo microplate reader (Revvity Health Sciences) with excitation at 530 nm and emission at 590 nm. Fluorescence values were corrected by subtracting background signals and normalized to the control group, which was set at 100%.

### 2.7 ROS Detection

ROS levels were quantified using the Total ROS/Superoxide Detection Kit (ROS‐ID, 51010, Enzo Biochem, Farmingdale, NY, USA), following prior optimization to determine the ideal concentration of pyocyanin as a positive control and the optimal incubation period with the fluorescent probe. The detection solution was prepared by diluting the Oxidative Stress Detection reagent in NIM to a final concentration of 0.12 nM. Pyocyanin (final concentration: 100 µM) was added to positive control wells. The background was subtracted using wells without cells, containing only the detection solution. After washing with PBS, cells were incubated for 5 h in the dark. Fluorescence was read using the VICTOR Nivo reader with excitation at 480 nm and emission at 530 nm. Values were normalized to the control group, considered as 100%.

### 2.8 Immunocytochemistry

Immunostaining was performed at two distinct stages to evaluate both neural induction in NSCs and the protein expression profile after serum treatment. Neural induction was confirmed by the expression of characteristic NSC markers, including SOX1, SOX2, PAX6, NESTIN, and FOXG1. In the serum treatment experiments, after 72 h of exposure, the expression of the same markers, except FOXG1, was re‐evaluated, along with additional proteins such as Caspase‐3, NeuN, p53, and Ki‐67. Passage characterization experiments and serum treatment assays were conducted as independent experimental frameworks with distinct biological objectives and culture conditions.

For staining, cells were seeded in 96‐well plates coated with Geltrex, allowed to adhere for 24 h, and then cultured for 72 h in the presence of serum. Following fixation with 4% paraformaldehyde for 10 min and PBS washing, cells were blocked with a solution containing 3% bovine serum albumin and 0.1% Triton X‐100 for 1 h. Primary antibodies (Table 3) were diluted according to the manufacturer's instructions and incubated for 16 h at 4°C in a humid chamber. After washing, secondary antibodies were applied for 2 h at room temperature in the dark. Nuclear staining was performed using Hoechst (Invitrogen, H1398, Life Technologies) diluted in PBS. Fluorescence images acquired with Nikon Eclipse Ti‐S and EVOS (M3000) microscopes were analyzed using ImageJ software (Schneider et al. 2012).

**TABLE 3:** Primary and secondary antibodies used in immunocytochemistry, including dilutions, supplier details, and biological functions of the target proteins.

Anti‐body	Specificity and description	Dilution	Commercial specification	Function
**Primary**	Anti‐SOX1 (polyclonal, goat, IgG)	1:300	R&D Systems, AF3369, Inc. a Bio‐Techne Brand, Minneapolis, USA	Neuroectoderm induction; specification of neural precursors (Pevny et al. 1998)
	Anti‐SOX2 (monoclonal, rabbit)	1:400	Cell Signaling Technology, 3579, Danvers, USA	Regulates pluripotency, neurodevelopment, and anterior neural progenitor identity (Tobias et al. 2025; S. Zhang et al. 2019)
	Anti‐PAX6 (monoclonal, mouse, IgG1)	1:200	Invitrogen, MA1109, Life Technologies, Burlington, Canada	Modulates neural gene expression and supports NSC multipotency and proliferation (Sansom et al. 2009; Xu et al. 2021)
	Anti‐NESTIN (monoclonal, mouse, IgG1)	1:200	Invitrogen, MA1110, Life Technologies, Burlington, Canada	Promotes NSC self‐renewal, differentiation, and cytoskeletal dynamics during migration (Di et al. 2014; Bernal and Arranz 2018)
	Anti‐FOXG1 (monoclonal, rabbit)	1:400	Cell Signaling Technology, 29642, Danvers, USA	Inhibits astrogliogenesis and stimulates proliferation of forebrain‐derived cells (Hettige et al. 2022; Bose et al. 2025)
	Anti‐CleavedCASPASE3 (monoclonal, rabbit)	1:400	Cell Signaling Technology, 9664, Danvers, USA	Activated by caspases and Atf3, involved in mitochondrial apoptosis, neurogenesis, and NSC homeostasis (Brentnall et al. 2014; Rosa et al. 2024)
	Anti‐NEUN (monoclonal, mouse)	1:1000	Cell Signaling Technology, 94403, Danvers, USA	Post‐mitotic neuronal marker; associated with terminal differentiation (Mullen et al. 1992; Gusel'nikova 2015)
	Anti‐P53 (monoclonal, mouse)	1:500	Cell Signaling Technology, 2524, Danvers, USA	Tumor suppressor involved in regulation of genes controlling apoptosis, senescence, and neuronal differentiation (Grigoreva and Glazova 2013; Y. Xiong et al. 2020)
	Anti‐KI67 (polyclonal, goat, IgG)	1:100	Santa Cruz Biotechnology Inc, sc‐7844, Dallas, EUA	Participates in cell proliferation, chromatin organization, and ribonucleoprotein coating of condensed chromosomes during mitosis (Sun and Kaufman 2018)
**Secondary**	Alexa Fluor 594 – goat anti‐Mouse IgG—Ex: 590 nm; Em: 618 nm	1:1000	Invitrogen, A11032, Life Technologies, Burlington, Canada	Fluorescent dye
	Alexa Fluor 488 – goat anti‐Mouse IgG—Ex: 499 nm; Em: 520 nm	1:1000	Invitrogen, A28175, Life Technologies, Burlington, Canada	Fluorescent dye
	Alexa Fluor 546 – donkey anti‐Goat IgG—Ex: 561 nm; Em: 572 nm	1:1000	Invitrogen, A11056, Life Technologies, Burlington, Canada	Fluorescent dye
	Alexa Fluor 594 – goat anti‐Rabbit IgG—Ex: 590 nm; Em: 618 nm	1:1000	Invitrogen, A11012, Life Technologies, Burlington, Canada	Fluorescent dye

Images were acquired under identical settings (exposure time, gain, contrast, and microscope configuration) to ensure comparability across groups. Fluorescence intensity was measured across the entire field of view without selecting individual cells. Threshold adjustments in ImageJ were applied to accurately reflect signal intensity. Hoechst‐stained nuclei were quantified using the *Binary—Watershed* algorithm combined with particle analysis, with minimum and maximum nuclear sizes defined. The ratio of fluorescence intensity to cell number was used as the primary parameter. The resulting values were tabulated and used for statistical comparison among the experimental groups.

### 2.9 Morphological Analyses

Cells were subjected to morphological analyses after neural induction and following treatment with the different serum samples. For post‐neural induction comparisons, cells were maintained in culture for 24 h and, following serum treatments, were cultured for 48 h prior to image acquisition for morphological evaluation. Morphological parameters, including cell circularity, mean branch length, and mean nuclear area, were quantified using ImageJ software (Schneider et al. 2012). For circularity analysis, bright‐field images were first subjected to automatic brightness and contrast adjustment, then background subtraction, Gaussian Blur filtering, threshold adjustment, segmentation of cells via watershed, and finally, analyze particles. For the mean branch length, immunocytochemistry images stained for NESTIN (a cytoskeletal marker) were processed by automatic brightness and contrast adjustment, followed by Gaussian Blur filtering, threshold definition, binarization, skeletonization using the Skeletonize plugin, and analysis via the Analyze Skeleton plugin. Nuclear size was quantified as mean nuclear area (µm^2^) from Hoechst‐stained images acquired in parallel experiments using calibrated, thresholded, and segmented images analyzed with the analyze particles function. The obtained parameters were then used for quantitative comparisons between experimental groups.

### 2.10 Statistical Analyses

Each serum sample was tested in triplicate (*n* = 6 per group). Data analysis was performed using GraphPad Prism software, version 8.0.1 (GraphPad Software, San Diego, CA, USA). Data distribution was initially assessed for normality. For comparisons between two groups, an unpaired Student's *t*‐test was applied for parametric data, and the Mann–Whitney test was used for non‐parametric data. For comparisons involving more than two groups, one‐way ANOVA followed by Tukey's post hoc test was used for parametric data, while the Kruskal–Wallis test followed by Dunn's post hoc test was applied for non‐parametric data. For experiments with two independent variables, two‐way ANOVA with Tukey's post hoc test was conducted. Statistical significance was set at *p* ≤ 0.05.

For metabolomic data, values were log_2_‐transformed before analysis. Differential abundance was assessed using log_2_ fold change and –log_10_‐transformed *p* values visualized in a volcano plot. Only metabolites detected in 100% of at least one group were included. Multiple testing correction was applied using the two‐stage Benjamini–Krieger–Yekutieli FDR procedure (*Q* = 1%).

### 2.11 Use of AI Tools

ChatGPT (OpenAI, San Francisco, CA, USA) was used solely for language editing. All scientific content, data interpretation, and conclusions were generated exclusively by the authors.

## 3 Results

### 3.1 Validation of Neural Induction by RT‐qPCR, Immunocytochemistry, and Morphological Analyses

**FIGURE 2:**
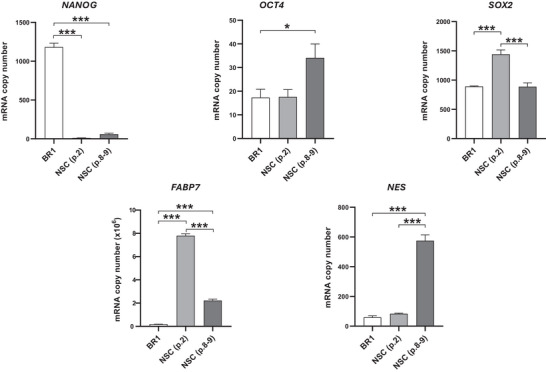
mRNA levels of pluripotency and neural lineage genes in BR1, NSC (p.2), and NSC (p.8–9). NANOG was most expressed in BR1, OCT4 in NSC (p.8–9), SOX2 in NSC (p.2), FABP7 in NSC (p.8–9), and NESTIN in NSC (p.2). [BR1, white bars; NSC (p.2), medium gray; NSC (p.8–9), dark gray]. Data are presented as mean ± SEM. *p < 0.05, ***p < 0.001. (p.2) passage 2, (p.8–9) passages 8 and 9. One‐way ANOVA with Tukey's post hoc test. *n* = 3 samples per group.

RT‐qPCR and immunocytochemistry analyses were employed to assess gene and protein expression of markers related to pluripotency and neural commitment across experimental groups. RT‐qPCR results (Figure 2; Table S1) revealed that *NANOG* expression was highest in the BR1 line and markedly reduced in NSCs regardless of passage. *OCT4* showed increased expression in NSCs at later passages (8–9). *SOX2* expression peaked at Passage 2, with no significant differences between BR1 and passages 8–9. For neural‐related genes, *FABP7* was detected in both passages, being more abundant in Passage 2, while *NESTIN* expression was higher in Passages 8–9.

**FIGURE 3:**
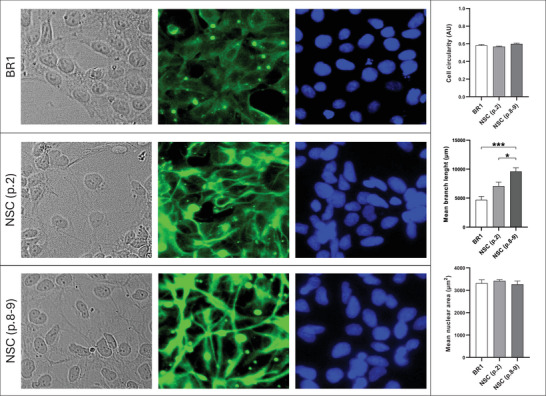
Morphological analysis of BR1 and NSC cells at different passages. No statistically significant differences were observed in cell circularity and mean nuclear area among groups. However, an increase in mean skeleton length was detected in NSCs at passages 2 and 8–9, with the highest values observed at passage 8–9. [BR1, white bars; NSC (p.2), medium gray; NSC (p.8–9), dark gray]. Data are presented as mean ± SEM. *p < 0.05, **p < 0.01. (p.2) passage 2, (p.8–9) passages 8 and 9. One‐way ANOVA with Tukey's post hoc test. *n* = 6 images per group.

**FIGURE 4:**
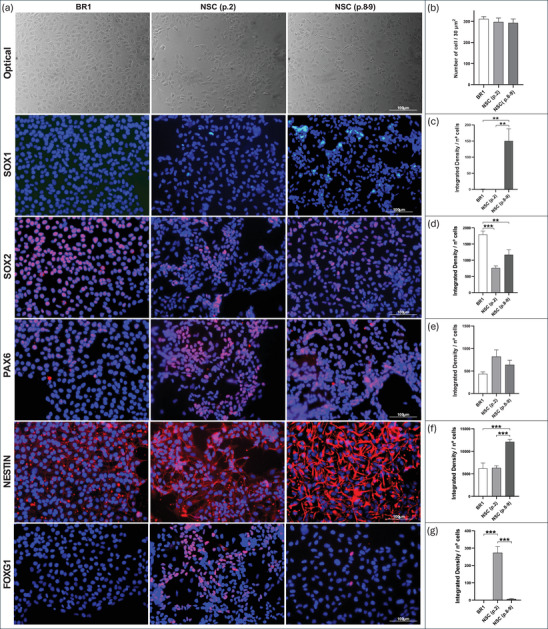
Representative immunocytochemistry images of BR1, NSC (p.2), and NSC (p.8–9), highlighting the expression of NSC‐associated proteins and corresponding quantification graphs. Quantitative analyses were performed within the same passage‐specific experimental framework, with fluorescence intensities normalized to total cell number. (**a**) Brightfield images show cell morphology; fluorescence images indicate marker expression. (**b**) Mean cell counts per field were similar across groups. (**c**) SOX1 labeling was most intense in NSCs (p.8–9). (**d**) SOX2 labeling was higher in BR1 compared to NSC (p.2). (**e**) PAX6 was expressed in all groups, with no statistical differences. (f) NESTIN expression was strongest in NSCs (p.8–9). (**g**) FOXG1 was predominantly expressed in NSCs (p.2). [BR1, white bars; NSC (p.2), medium gray; NSC (p.8–9), dark gray]. Data are shown as mean ± SEM. *p < 0.05, **p < 0.01, ***p < 0.001. (p.2) passage 2, (p.8–9) passages 8 and 9. One‐way ANOVA with Tukey's post hoc test. *n* = 5 images per group. Scale bars: 100 µm.

Morphological analyses (Figure 3; Table S1) of BR1 and NSCs at different passages revealed a progressive increase in mean branch length. NSCs at Passage 2 showed higher values compared with BR1, and the greatest values were observed at Passages 8–9, indicating longer cytoplasmic processes following induction. Cell quantification in the immunocytochemistry assays revealed comparable mean values across groups (Figure 4a,b). In addition, the staining patterns confirmed a stage‐dependent modulation of neural markers. SOX1 showed stronger staining at Passages 8–9 (Figure 4c; Table S1), while SOX2 was more intense in BR1 (Figure 4d). PAX6 was detected in all groups (Figure 4e). NESTIN exhibited stronger labeling at Passages 8–9 (Figure 4f), whereas FOXG1 predominated at Passage 2 (Figure 4g). Together, these observations indicate dynamic changes in gene and protein expression throughout successive stages of NSC differentiation.

PSC‐derived neural cultures at this stage are heterogeneous and comprise both NSCs and early neural progenitors (Shi et al. 2012), which differ in self‐renewal capacity and lineage restriction (Florio and Huttner 2014; Obernier and Alvarez‐Buylla 2019). For this reason, the term “NSCs” is used operationally to describe the mixed precursor population present under our culture conditions.

### 3.2 Serum Metabolomic Differences Between NO and OB Donors

**FIGURE 5:**
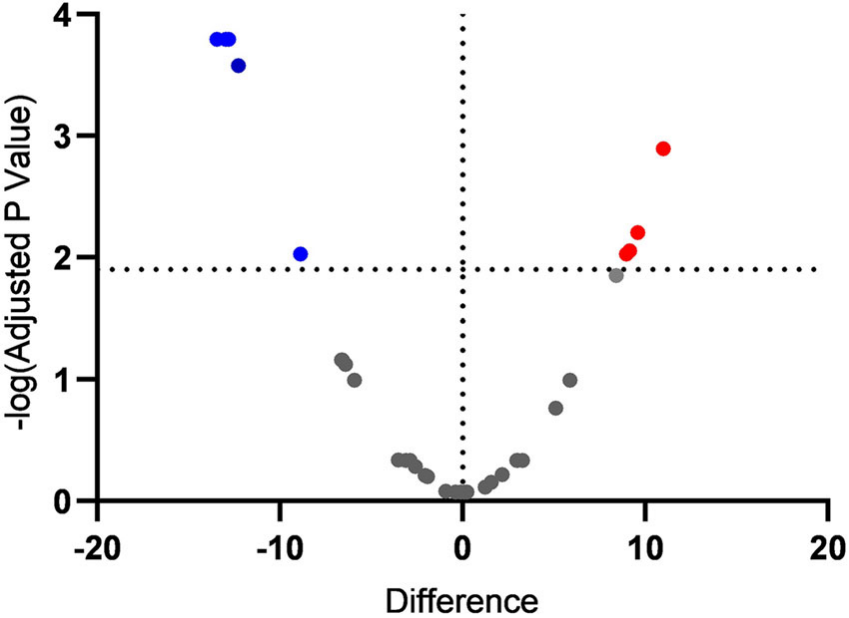
Volcano plot of differentially abundant serum metabolites in OB versus NO women. The *x*‐axis represents the log_2_ difference in serum metabolite abundance between OB and NO groups, and the *y*‐axis shows the –log_10_ FDR‐adjusted *p* value. Metabolites above the significance threshold (horizontal dotted line; FDR = 0.1, –log_10_ ≈ 1.9) are highlighted in color: Red for metabolites increased in OB and blue for those decreased. Gray points indicate non‐significant metabolites. The vertical line marks no difference between groups. Metabolites differentially present are presented in Table 4.

Metabolomic analysis identified 40 out of 337 serum metabolites as differentially abundant in women with obesity compared with eutrophic controls (Figure 5). Among these, only nine reached statistical significance (Table 4), and six were highlighted in this study due to their relevance to metabolic alterations commonly associated with obesity.

**TABLE 4:** Metabolites with altered serum concentrations in OB compared with NO women.

Metabolite	Relative abundance vs. NO (↑/↓)	*p*‐value	*Q*‐value (corrected *p*‐value)
19‐hydroxy‐nonadecanoic acid	↑	0.000005	0.000160
PS(P‐20:0/16:0)		0.000014	0.000160
(3S,5R,6R)‐3,5‐dihydroxy‐6,7‐didehydro‐5,6‐dihydro‐12′‐apo‐beta‐caroten‐12′‐al		0.000031	0.000264
6‐Oxocativic acid		0.002458	0.009377
Pentadecyl acetate **(ND)**		0.000011	0.000160
2‐Hydroxyenterodiol	↓	0.001798	0.008820
DG(15:0/18:1(11Z)/0:0)		0.000185	0.001271
Glycerol tripropanoate **(ND)**		0.002238	0.009377
(3alpha,5beta,11beta,17beta)‐9‐Fluoro‐17‐methylandrostane‐3,11,17‐triol **(ND)**		0.001089	0.006231

*Note*: *p* values were adjusted for multiple testing using the two‐stage Benjamini–Krieger–Yekutieli false discovery rate (FDR) procedure (*Q* = 1%). Arrows indicate the direction of change relative to the NO group (↑ increase; ↓ decrease). ND denotes metabolites not discussed in detail.

Metabolites present at higher concentrations in OB serum included: (1) PS(P‐20:0/16:0), (2) 19‐hydroxynonadecanoic acid, (3) (3S,5R,6R)‐3,5‐dihydroxy‐6,7‐dihydro‐12′‐apo‐β‐carotene‐12′‐al, and (4) 6‐Oxocativic acid. Those present at lower concentrations were: (5) diacylglycerol (15:0/18:1), and (6) 2‐hydroxyenterodiol.

Three additional statistically significant metabolites—pentadecyl acetate, (3α,5β,11β,17β)‐9‐fluoro‐17‐methyl‐androstane‐3,11,17‐triol, and glycerol tripropanoate—were excluded from interpretation. These compounds have no known scientific documentation or established relevance in humans. In untargeted metabolomics, many detected features may reflect artifacts, contaminants, or poorly annotated molecules rather than true endogenous metabolites (Gertsman and Barshop 2018; Chaleckis et al. 2019). Therefore, only metabolites with reliable annotation and plausible biological relevance were considered for interpretation to avoid misleading conclusions.

### 3.3 Human Serum‐Induced Changes in NSC Viability, Oxidative Stress, and PI Uptake

**FIGURE 6:**
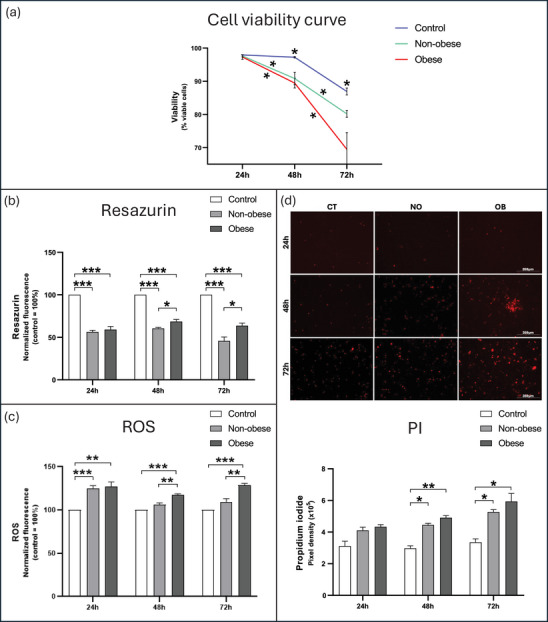
Viability curve, relative fluorescence assessment of resazurin reduction, ROS production, and PI incorporation in CT, NO, and OB groups after 24, 48, and 72 h of treatment with human sera. Fluorescence values for resazurin reduction and ROS production were normalized to the control group (CT = 100%). (a) Viability curve of NSCs at 24, 48, and 72 h. Both serum‐treated groups showed reduced viability compared with CT at 48 and 72 h, with progressive declines in the NO and OB groups over time. (b) Lower bars in the NO and OB groups indicate reduced resazurin conversion and, therefore, decreased metabolic activity. Both NO and OB groups showed a significant reduction in resazurin metabolism at all time points, with a more pronounced decline in the NO group at 48 and 72 h. (c) ROS production significantly increased in both serum‐treated groups at 24 h, with the OB group displaying the highest fluorescence levels at 48 and 72 h. (d) Representative fluorescence images illustrate cellular labeling in each experimental group and time point; pixel density graphs show significantly higher PI incorporation in both serum‐treated groups. (a: CT, blue line; NO, green line; OB, red line). (b–d: CT, white bars; NO, light gray; OB, dark gray). Data presented as mean ± SEM. *p < 0.05, **p < 0.01, ***p < 0.001. (CT) control, (NO) non‐obese, (OB) obese. Two‐way ANOVA with Tukey's multiple comparisons. *n* = 6 donors per group. Scale bars: 359 µm.

These metabolite alterations, together with additional serum components not examined here, appear to influence NSCs. This effect was first evident in the viability assay (Figure 6a; Table S2; Table 5). No differences were detected at 24 h. At 48 and 72 h, both serum‐treated groups showed reduced viability compared with the CT group, with no statistical differences between them. In the longitudinal analysis, the NO and OB groups showed a consistent decline across all time points.

**TABLE 5:** Comparative analysis of cell viability, metabolic activity, ROS production, and PI labeling across exposure times in the NO and OB groups.

Experimental analysis	Group	Two‐way ANOVA (interaction *p*‐value)	Post‐hoc comparisons (*p*)	
**Cell viability**	**NO**	0.027	24 vs. 48h	↓; *p* = 0.034
			48 vs. 72h	↓; *p* = 0.039
			24 vs. 72h	↓; *p *<0.001
	**OB**		24 vs. 48h	↓; *p* = 0.010
			48 vs. 72h	↓; *p* = 0.018
			24 vs. 72h	↓; *p* = 0.009
**Resazurin reduction**	**NO**	0.003	24 vs. 48h	Ns; *p* = 0.072
			48 vs. 72h	↓; *p* = 0.038
			24 vs. 72h	Ns; *p* = 0.250
	**OB**		24 vs. 48h	↑; *p* = 0.013
			48 vs. 72h	Ns; *p* = 0.218
			24 vs. 72h	Ns; *p* = 0.452
**ROS detection**	**NO**	0.002	24 vs. 48h	↓; *p* = 0.018
			48 vs. 72h	Ns; *p* = 0.441
			24 vs. 72h	Ns; *p* = 0.128
	**OB**		24 vs. 48h	Ns; *p* = 0.183
			48 vs. 72h	↑; *p* = 0.017
			24 vs. 72h	Ns; *p* = 0.969
**PI tagging**	**NO**	0.233	24 vs. 48h	Ns; *p* = 0.380
			48 vs. 72h	Ns; *p* = 0.001
			24 vs. 72h	↑; *p* = 0.032
	**OB**		24 vs. 48h	↑; *p* = 0.041
			48 vs. 72h	Ns; *p* = 0.246
			24 vs. 72h	Ns; *p* = 0.062

*Note*: Data were analyzed using two‐way ANOVA followed by Tukey's post hoc test; overall ANOVA and pairwise comparison *p* values are shown. Arrows indicate the direction of change (↓ decrease; ↑ increase), and Ns denotes non‐significant differences.

Consistently, functional assays confirmed the impact of serum exposure. After 24 h, both serum‐treated groups showed diminished resazurin reduction (Figure 6b; Table S2), increased ROS levels (Figure 6c; Table S2), and higher PI incorporation compared to controls, with no distinction between NO and OB (Figure 6d; Table S2). At 48 h, the reduction of resazurin remained significantly lower, especially in NO, whereas ROS accumulation was more prominent in OB. After 72 h, metabolic activity further declined, predominantly in the NO group, whereas ROS levels remained elevated in OB.

In the intra‐group analysis (Table 5), the lowest resazurin reduction in the NO group was observed at 72 h, whereas in the OB group, the most pronounced reduction occurred at 48 h. In terms of ROS, the NO group showed a decrease from 24 to 48 h that remained stable thereafter, while the OB group exhibited an increase from 48 to 72 h. PI incorporation showed an increase between 24 and 48 h only in the OB group.

### 3.4 Morphological and Protein Expression Changes Associated With Neurogenesis, Proliferation, and Apoptosis in NSCs Treated With Serum From NO and OB Donors

NSCs behavior depends on the coordinated regulation of proliferation, metabolism, and differentiation (Scandella et al. 2023; Semkova et al. 2022). Circulating factors associated with obesity may disrupt these processes by affecting mitochondrial function, redox balance, and lineage specification (Ramosaj et al. 2021). To investigate this, morphological analyses and immunocytochemistry were performed following treatments to determine whether serum conditions induced measurable changes in NSC morphology and in markers of neural identity, proliferation, stress, and apoptosis. This approach provides an initial framework for understanding how obesity‐associated factors may influence neural progenitor function.

**FIGURE 7:**
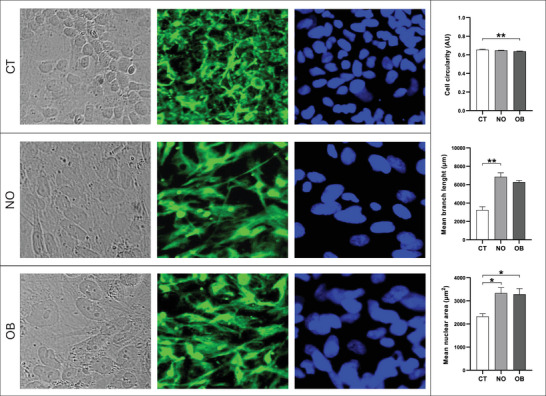
Morphological analysis of cells after treatment with the different sera. Cells treated with serum from OB donors exhibited a slightly less circular morphology, whereas those treated with serum from NO donors showed a greater mean skeleton length. In addition, the CT group displayed a smaller mean nuclear area compared with the NO and OB groups. [CT, white bars; NO, medium gray; OB, dark gray]. Data are presented as mean ± SEM. *p < 0.05, **p < 0.01. (CT) control, (NO) non‐obese, (OB) obese. Cell circularity and mean nuclear area: One‐way ANOVA with Tukey's post hoc test; Mean branch length: Kruskal–Wallis with Dunn. *n* = 6 donors per group (1 image per donor).

Cells treated with the sera exhibited distinct morphological profiles (Figure 7; Table S3). The CT group showed slightly higher circularity compared with the OB group. NSCs in both the NO and OB groups displayed longer cytoplasmic processes, with the NO group showing a statistically significant increase relative to the CT group. In addition, nuclear area was increased in cells treated with NO and OB serum compared with CT, with no significant difference between the two treatments.

**FIGURE 8:**
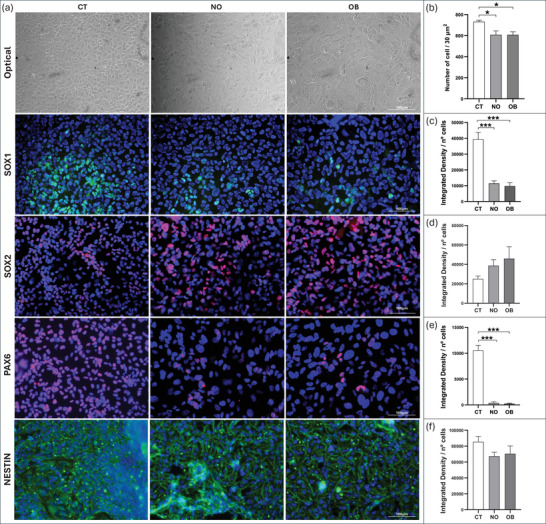
Immunocytochemical analysis of neural markers in CT, NO, and OB groups with corresponding quantifications. Quantitative analyses were performed within the same experimental set, with all groups analyzed at identical culture durations and treatment times. Fluorescence intensities were normalized to total cell number. (a) Representative immunofluorescence images for each analyzed protein. (b) Quantification of cell number, showing comparable mean values between the NO and OB groups. (c) Decreased SOX1 expression in NO and OB groups. (d) SOX2 detection across all groups, without significant differences. (e) Reduced PAX6 labeling in serum‐treated groups. (f) Comparable NESTIN expression across groups. (CT, white bars; NO, medium gray; OB, dark gray). Data expressed as mean ± SEM. *p < 0.05, **p < 0.01, ***p < 0.001. (CT) control, (NO) non‐obese, (OB) obese. One‐way ANOVA with Tukey. *n* = 6 donors per group (3 images per donor). Scale bars: 100 and 359 µm.

The average number of cells per field was approximately 17% higher in the control group compared with the NO and OB groups, which showed similar values (Figure 8a,b; Table S3). Regarding protein expression, SOX1 labeling was reduced in the groups treated with NO and OB serum (Figure 8c). SOX2 showed increased labeling in the treated groups, particularly in OB, although no statistical differences were detected (Figure 8d). PAX6 expression was highest in the control group, with reduced levels in both treated groups (Figure 8e). NESTIN expression remained stable across all groups (Figure 8f).

**FIGURE 9:**
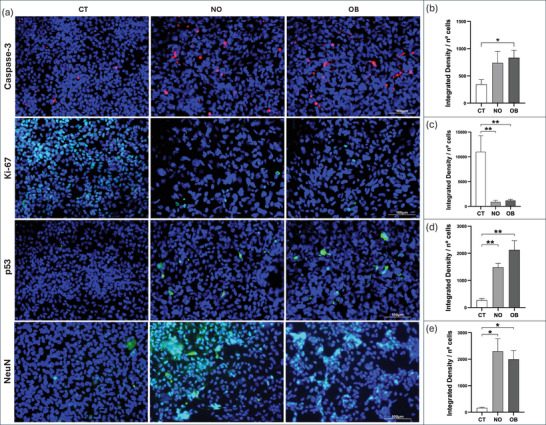
Immunofluorescence analysis of additional markers in experimental groups. Quantification was conducted under identical experimental conditions across groups, with fluorescence signals normalized to total cell number. (a) Representative fluorescence images for each marker. (b) Quantitative analysis indicating increased Caspase‐3 expression in the OB group. (c) Decreased Ki‐67 detection following serum exposure. (d) Elevated P53 levels in NO and OB groups. (e) Enhanced NEUN expression in serum‐conditioned cells. (CT, white bars; NO, medium gray; OB, dark gray). Data shown as mean ± SEM. *p < 0.05, **p < 0.01, ***p < 0.001. (CT) control, (NO) non‐obese, (OB) obese. Caspase‐3, NeuN: Kruskal–Wallis with Dunn; Ki‐67, p53: one‐way ANOVA with Tukey. *n* = 6 donors per group (3 images per donor). Scale bars: 100 and 359 µm.

Quantitative analysis revealed increased Caspase‐3 expression in the OB group compared with the CT group (Figure 9a,b; Table S3). Ki‐67 expression decreased following treatment (Figure 9c). P53 staining intensity was elevated in both the NO and OB groups relative to the control (Figure 9d), mirroring the pattern observed for NeuN (Figure 9e). However, when comparing the NO and OB groups directly, no significant differences were detected for any of the proteins analyzed in this study.

## 4 Discussion

The combined analysis of RT‐qPCR and immunocytochemistry data robustly confirmed the differentiation of ESCs into NSCs. Variations observed across different NSC passages suggest a time‐dependent modulation of markers related to proliferation and neural identity, indicating a gradual consolidation of the neural phenotype over time, at least until Passages 8–9. Genes and protein markers associated with pluripotency—*NANOG*, *OCT4*, and *SOX2*—were expressed in the BR1 ESCs (Figure 2, Table S1), reflecting their capacity for trilineage differentiation (Wernig et al. 2007; S. Zhang and Cui 2014; Zhou et al. 2016; W. Zhang et al. 2016; L. Xiong et al. 2022). Cells undergoing neural commitment or already specified toward the neural lineage—capable of generating neurons, astrocytes, and oligodendrocytes—retain both multipotency and self‐renewal potential (Allen 2008; Adachi et al. 2010; S.‐H. Lee et al. 2010; Bertolini et al. 2016; Zhou et al. 2016; Tobias et al. 2025). Accordingly, continued expression of genes sustaining these properties, such as *SOX2* and *OCT4*, was observed in our data.

NSC selection was performed through the isolation of well‐defined rosettes using microscopic observation and a specific detachment reagent, and the structures exhibited clear NSC morphology (Figure 1) (Whye et al. 2023). The persistence of *OCT4* and *NANOG* expression in Passages 8–9 (Figure 2; Table S1) was unexpected and may represent a particular feature of the BR‐1 line. Nonetheless, confirmation of complete pluripotency loss would require terminal differentiation assays, which we recognize as a limitation in interpreting differentiation and proliferation outcomes.

Morphologically, NSCs exhibited cellular projections (Figure 3; Table S1) consistent with a neural identity (Lü et al. 2004; Bakhru et al. 2011). NSC identity was confirmed by the expression of *NESTIN* (Wiese et al. 2004; Thomson et al. 2021), *FABP7* (Arai et al. 2005; Y. Lee et al. 2024), *SOX1*, *PAX6* (Kanwore et al. 2021), and FOXG1 (Ferguson et al. 2024; Bose et al. 2025) (Figures 2 and 4; Table S1). These findings collectively support the occurrence of progressive neural differentiation, with passage‐dependent changes in marker expression, suggesting a dynamic process governing both neural identity and proliferation. This characterization allowed us to explore the behavior of NSCs under distinct microenvironments: Serum from NO women of reproductive age and serum from age‐matched women with obesity.

In vitro models have been widely used to mimic human obesity by modifying the cellular environment (Turner et al. 2014; Zagotta et al. 2015). These approaches often rely on metabolic or hormonal manipulation, such as multi‐tissue systems that recreate crosstalk between adipose and metabolic organs (Vinci et al. 2012), or models that induce obesogenic conditions through altered pH, hormones, cytokines, or glucose (Ghanemi et al. 2022). Adipocyte hypertrophy can also be triggered with high glucose and insulin (Qiao et al. 2020). While informative, these models simulate obesity indirectly. In contrast, our study employs human serum as a physiological source of metabolic signals, allowing a direct comparison of how serum from individuals with or without obesity influences NSC behavior.

Human serum is a complex biological fluid composed of proteins, metabolites, ions, and cytokines, whose application in cell culture can modulate proliferation and differentiation (Örnemark et al. 1999; Pisciotta et al. 2012). Particularly, serum from individuals with obesity presents notable compositional changes, such as increased levels of metals, inflammatory proteins, and pro‐oxidant metabolites, alongside decreased antioxidant compounds (P. Wang et al. 2016; Karkhaneh et al. 2017). These alterations can impact cell behavior, affecting viability, metabolism, and proliferation.

Given these context‐specific differences, preserving the native composition of the sera was essential for accurately modeling their biological effects. The serum used in this study was therefore not heat‐inactivated to maintain physiologically relevant components altered in obesity, including lipids, inflammatory mediators, and complement proteins. Complement factors such as C3, C4, factor B, factor D, and the activation fragments C3a and C5a are elevated in obesity and contribute to its chronic pro‐inflammatory and metabolic profile (Shim et al. 2020; Phieler et al. 2013). Heat inactivation would remove or denature these elements and produce a serum that no longer reflects in vivo conditions. Complement also has established roles in neural systems, influencing neurodevelopment, synaptic remodeling, ROS production, calcium signaling, and caspase‐dependent apoptosis (Rahpeymai et al. 2006; Stevens et al. 2007; Mahajan et al. 2016; Brennan et al. 2012). Thus, complement activity may contribute to the differences observed between OB and NO serum. Heat treatment also alters cytokine stability and complement‐dependent signaling, creating non‐physiological effects (Fante et al. 2021). Because both serum groups were processed in the same way, the differences in NSCs reflect biological variation between donor groups.

Metabolomic analysis revealed significant differences in the serum metabolic profiles of women with obesity compared to NO controls (Figure 5, Table 4). Among the upregulated metabolites, PS(P‐20:0/16:0) belongs to a phospholipid class associated with membrane structure and signaling (Schiller and Arnold 2002; Anjani et al. 2015; Hinkley and Coen 2020). In addition, 19‐hydroxynonadecanoic acid, part of the ω‐hydroxy long‐chain fatty acid group, typically linked to structural and signaling roles in lipid systems, was also elevated (Wertz 2018; Pikó et al. 2021; NCBI 2025c). These molecules represent oxidized lipid species that can modulate intracellular oxidative tone and influence basal ROS levels (Ayala et al. 2014), potentially contributing to the higher ROS detected in NSCs exposed to OB serum. Such metabolic alterations are consistent with adaptive responses to oxidative stress and low‐grade inflammation characteristic of obesity (Pamplona 2008; Mika and Sledzinski 2017; Anjos et al. 2019) and likely reflect cytokine‐driven lipid remodeling associated with adipose tissue dysfunction (Kang et al. 2013; Alsharari et al. 2017).

(3S,5R,6R)‐3,5‐Dihydroxy‐6,7‐dihydro‐12′‐apo‐β‐caroten‐12′‐al, a carotenoid‐derived metabolite that belongs to a chemical group characterized by antioxidant and redox‐modulating activity (Raju et al. 2021; NCBI 2025b), was also increased, suggesting enhanced lipid peroxidation (Harari et al. 2020; Q. W. Zhang et al. 2023; Yan et al. 2023). 6‐Oxocativic acid, a diterpenoid identified in *Cistus ladanifer* and representing a class of plant‐derived molecules with no established function in humans (Alías et al. 2012; NCBI 2025a), was likewise elevated.

Conversely, two metabolites showed lower concentrations: DG (15:0/18:1) and 2‐hydroxyenterodiol. Diacylglycerol (15:0/18:1) is part of a group of intermediates involved in triglyceride metabolism and intracellular signaling (Rudkowska et al. 2005; Roe et al. 2018). 2‐Hydroxyenterodiol belongs to a class of hydroxylated metabolites with limited functional evidence, although related compounds have been associated with energy metabolism (Tofovic et al. 2001). These reductions may indicate decreased triglyceride synthesis and diminished hormonal protection (Kolczynska et al. 2020).

Because the differentially abundant metabolites identified in this study have not been functionally characterized, their interpretation relies on properties shared within their broader chemical classes. No studies have assessed their individual roles in human physiology or their effects on neurogenesis, and their potential materno‐fetal transfer also remains unknown. Nonetheless, several related classes of small lipophilic molecules, such as specific fatty acids, phospholipids, diterpenoids, and phenolic compounds, are known to cross the placenta (Duttaroy 2009; Larqué et al. 2011; Roberts et al. 2014; Jensen et al. 2019). These gaps highlight the need for targeted studies to determine the physiological relevance and transferability of the metabolites identified here.

Under physiological conditions, developing brain cells are initially protected by the placental barrier and later by the BBB, both of which restrict the passage of maternal blood components. The placental barrier, for instance, limits the transfer of inflammatory cytokines such as IL‐1β, IL‐6, and TNF‐α, found at higher concentrations in individuals with obesity (Zaretsky et al. 2004; Esser et al. 2014), as well as high‐molecular weight metabolites like albumin (Prouillac and Lecoeur 2010). Although this protection reduces direct fetal exposure, barrier permeability can increase in conditions such as obesity (Kim et al. 2014; Kretschmer et al. 2020), thereby altering the transport of metabolites, inflammatory mediators, and other circulating factors into the developing brain (Haddad‐Tóvolli et al. 2023; Feng et al. 2024).

In parallel, NSCs are positioned away from direct serum contact within the early neuroepithelium, yet their behavior is influenced by endothelial‐derived cues as vascularization progresses (Paridaen and Huttner 2014; Tata and Ruhrberg 2018). Because the BBB remains immature during these stages, maternal serum components can reach the developing neural microenvironment through nascent vessels (Haddad‐Tóvolli et al. 2017; Saunders et al. 2012). Thus, although exposure to human serum represents an artificial scenario, it provides a means to assess systemic metabolic and inflammatory signals to which NSCs may be vulnerable during early development.

In our experiments, this exposure resulted in reduced cell viability accompanied by increased apoptosis, as indicated by elevated levels of markers such as Caspase‐3 (Figure 9b; Table S3) and PI (Figure 6d; Table S2) in both groups. Concomitantly, an increase in nuclear size was observed (Figure 7; Table S3) alongside a decline in proliferative capacity, as evidenced by reduced expression of Ki‐67 (Figure 9c; Table S3) and PAX6 (Figure 8e; Table S3), key regulators of NSC proliferation, differentiation, and migration (Arai et al. 2005; Sun and Kaufman 2018; Han et al. 2025). Although differentiation can reduce cell numbers over time, apoptosis typically proceeds rapidly once triggered, making it the more likely explanation for the reduction in viable cells observed in our models.

In both serum‐treated groups, Ki‐67 immunolabeling was reduced (Figure 9c; Table S3). p53 was simultaneously upregulated (Figure 9d; Table S3), consistent with its role in stress‐induced cell‐cycle arrest and differentiation (Hede et al. 2011; Fu et al. 2020; Y. Xiong et al. 2020). Progenitor markers such as SOX1 and PAX6 were also decreased (Figure 8c,e). These coordinated changes point to the involvement of broader stress‐response and cell‐cycle regulatory pathways. Beyond possible mTOR involvement (Romanyuk et al. 2024), stress‐activated mechanisms including the p53‐p21 axis (Meletis et al. 2006), oxidative and inflammatory signaling (Xue et al. 2025), and disruptions in developmental pathways such as Notch and BMP/TGF‐β (Lathia et al. 2008) likely converge to limit self‐renewal and promote early differentiation. Thus, the observed proliferation deficits and premature lineage commitment arise from the integrated action of multiple regulatory pathways.

We also observed increased expression of NeuN (Figure 9e), a neuronal differentiation marker, indicating that human serum may prompt early neural commitment at the expense of maintaining proliferative capacity (Englund et al. 2005; Alekseeva et al. 2015). However, because our marker panel did not include additional mature neuronal markers, the precise differentiation outcome cannot be determined, and establishing lineage identity was not within the scope of this study.

The observed changes, including reduced expression of PAX6, SOX1, and Ki‐67, alongside elevated p53 and NeuN (Figures 8 and 9; Table S3), suggest that exposure of NSCs to human serum disrupts the maintenance of the progenitor state, elicits cellular stress responses, and promotes early differentiation. However, when comparing the effects of NO and OB serum, we found that most cellular responses—including those related to apoptosis (Caspase‐3), proliferative potential (Ki‐67), SOX2, PAX6, NESTIN, p53, and NeuN—were similar between groups.

Although both serum‐treated groups showed reduced resazurin metabolism compared to the control, the OB group retained higher metabolic activity than the NO group (Figure 6b; Table S2). Cells treated with OB serum also exhibited increased ROS production, indicating a stronger functional impact of OB serum on NSCs (Figure 6c; Table S2). Resazurin indicates early metabolic impairment, whereas PI staining marks loss of membrane integrity, a late event in cell death (Präbst et al. 2017; Rieger et al. 2011). Thus, OB‐treated cells may preserve metabolic activity even as structural damage progresses.

The higher resazurin metabolism observed in the OB group at 48 and 72 h (Figure 6b) suggests that OB serum provides substrates that transiently support mitochondrial activity. This finding aligns with the enhanced metabolic pathways reported in cells from individuals with obesity (Mastrangelo et al. 2016; Katare et al. 2022). Elevated circulating lipids, particularly free fatty acids and triglycerides may promote mitochondrial β‐oxidation and biogenesis (Talari et al. 2023), thereby increasing NADH and FADH_2_ production and driving electron flux through the respiratory chain (Houten et al. 2016; Console et al. 2020). This enhanced redox activity may accelerate resazurin reduction initially, whereas prolonged lipid exposure can induce lipotoxicity, oxidative stress, and mitochondrial dysfunction (Hasan‐Olive et al. 2019).

Both serum‐treated groups exhibited an oxidative shift at 24 h; however, only the OB serum‐treated cells maintained elevated ROS levels at later time points (Figure 6b), indicating insufficient antioxidant capacity. This sustained oxidative stress aligns with mitochondrial overload and the chronic inflammatory environment characteristic of obesity (Monserrat‐Mesquida et al. 2021; Sindhu et al. 2018). Nevertheless, serum lipid composition and mitochondrial function were not directly assessed; therefore, these mechanistic interpretations should be considered with caution.

Although physiological levels of ROS play important roles in stem cell proliferation, survival, and differentiation, excessive ROS is well known to trigger oxidative stress (Sart et al. 2015). Experimental data indicate that changes in ROS‐related pathways or mitochondrial dysfunction are directly linked to NSC differentiation (W. Wang et al. 2011; Park et al. 2016), which supports our findings.

Our results demonstrate that bioactive components present in human serum disrupt NSC homeostasis by impairing differentiation dynamics, reducing cell viability, and increasing oxidative stress. Although exposure to serum from OB and NO women produced largely similar effects, serum from women with obesity consistently induced higher ROS levels, indicating subtle yet reproducible differences in redox regulation. These findings suggest that obesity‐related systemic alterations may manifest as incremental changes rather than overtly distinct phenotypes in vitro, which may nonetheless be biologically relevant during early neurodevelopment, a period of heightened sensitivity to redox imbalance in NSCs. Collectively, our results indicate that obesity‐associated systemic features can exert additional, context‐dependent effects that warrant further investigation.

Metabolomic analysis identified nine metabolites that differed between OB and NO serum. Although their specific roles cannot be defined here, many are lipid‐derived or redox‐active and could influence oxidative balance and cellular homeostasis. These metabolites therefore represent promising targets for future studies investigating serum‐derived factors that affect NSC differentiation, viability, and redox regulation.

This study presents some limitations. The use of 10% serum may introduce non‐physiological cues that influence NSC responses, and lower concentrations should be explored in future work. In addition, the metabolomic analysis was descriptive and does not establish causal links between specific metabolites and the observed cellular effects.

## Author Contributions

**Phelipe Elias da Silva**: conception and design, performed all techniques, analysis of the results, manuscript writing. **Natássia Caroline Resende Corrêa**: conception and design, manuscript correction. **Natália Ferreira Silva**: donor selection, serum collection. **Carlos Ueira‐Vieira**: financial support, manuscript correction. **Hebreia Oliveira Almeida‐Souza**: performed metabolomic analysis of serum, conducted statistical analyses. **Mario Machado Martins**: performed metabolomic analysis of serum, organized study materials. **Tiara da Costa Silva**: performed metabolomic analysis of serum. **Renata Graciele Zanon**: conception and design, provision of study material, financial support, analysis of the results, manuscript correction.

## Funding

This work was supported by Minas Gerais State Research Support Foundation (FAPEMIG) codes APQ‐01483‐22 and CBB‐APQ‐03613‐17; National Council for Scientific and Technological Development (CNPq) code 403193/2022‐2; Coordenação de Aperfeiçoamento de Pessoal de Nível Superior—Brasil (CAPES) finance code 001.

## Conflicts of Interest

The authors declare no conflicts of interest.

## Supporting information




**Supplementary Figure 1**. Major signaling pathways and small molecules involved in the in vitro neuroectodermal differentiation of human PSCs.
For in vitro neural differentiation, EBs are maintained in NIM supplemented with specific small molecules. These include LDN193189, an inhibitor of the Bone Morphogenetic Protein (BMP) pathway, and SB431542, which inhibits Transforming Growth Factor beta (TGF‐β) signaling. The combined use of these inhibitors constitutes the dual SMAD inhibition strategy. Additional molecules required for efficient neural induction include XAV939, which attenuates Wnt signaling, and SU5402, an inhibitor of the Fibroblast Growth Factor (FGF) receptor (Whye et al. 2023).

**Supplementary Table 1**. Statistical results of RT‐qPCR, morphometric, and immunocytochemical analyses performed in BR1 cells and NSCs at passages 2 and 8–9.
**Supplementary Table 2**. Results of cell viability, resazurin reduction, ROS production, and PI staining analyses among the CT, NO, and OB groups at 24, 48, and 72 h.
**Supplementary Table 3**. Results of immunocytochemical and morphometric analyses in NSCs under CT conditions and following treatment with serum from NO and OB individuals.

## Data Availability

The data that support this study are available from the corresponding author upon reasonable request.
