# Supplementary figures and images for: Blood Serum From Obese Women Raises ROS Production by Neural Stem Cells
Source: Dev Neurobiol. 2026 Mar 17;86(2):e70021. doi: 10.1002/dneu.70021 (PMC12996446; doi:10.1002/dneu.70021)

## Dual SMAD inhibition

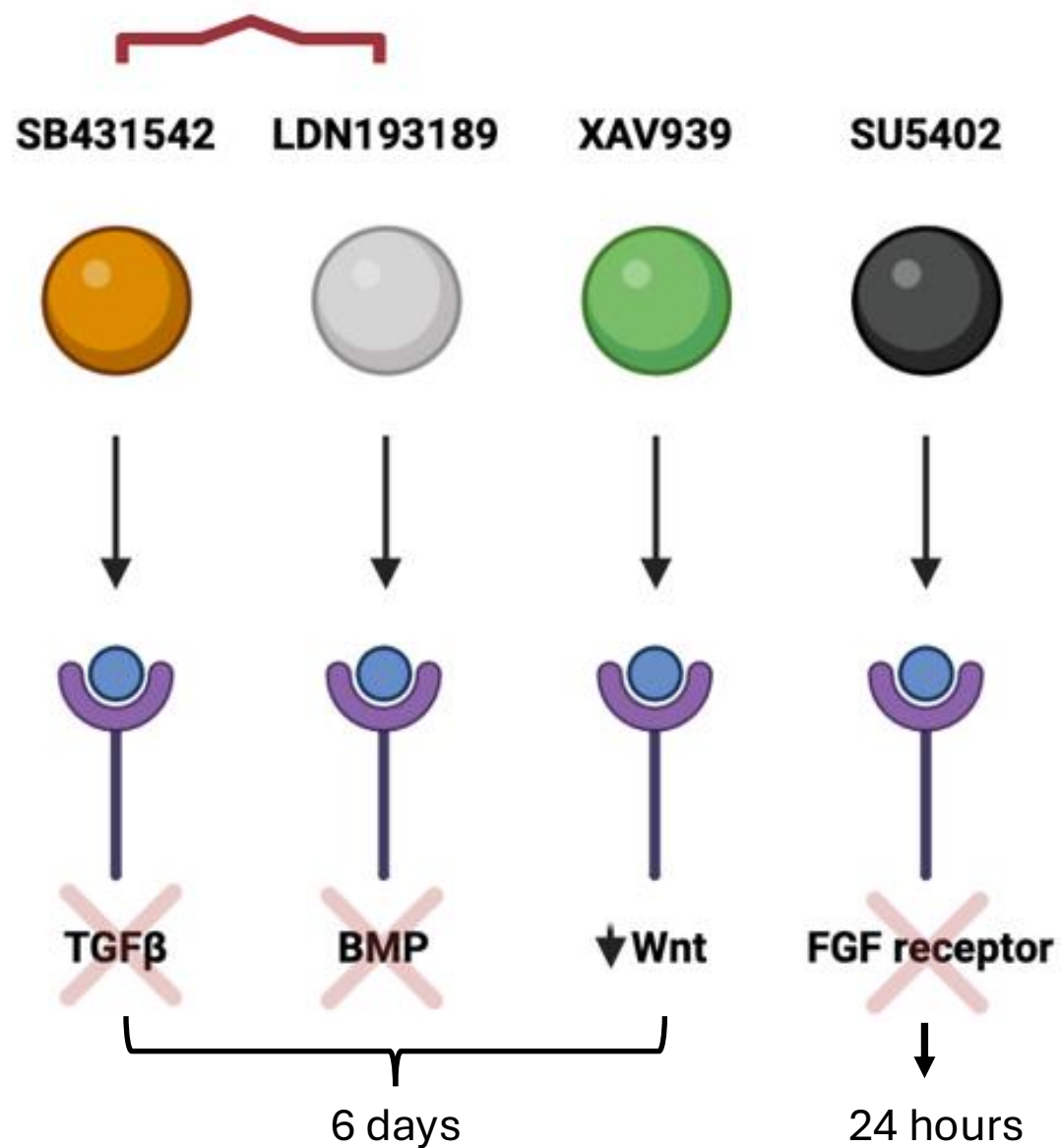

Supplement: Supplementary file 1 — Supplementary Figure 1. Major signaling pathways and small molecules involved in the in vitro neuroectodermal differentiation of human PSCs. For in vitro neural differentiation, EBs are maintained in NIM supplemented with specific small molecules. These include LDN193189, an inhibitor of the Bone Morphogenetic Protein (BMP) pathway, and SB431542, which inhibits Transforming Growth Factor beta (TGF‐β) signaling. The combined use of these inhibitors constitutes the dual SMAD inhibition strategy. Additional molecules required for efficient neural induction include XAV939, which attenuates Wnt signaling, and SU5402, an inhibitor of the Fibroblast Growth Factor (FGF) receptor (Whye et al. 2023). [file DNEU-86-0-s001.pdf]
