# Supplementary material for: Blood Serum From Obese Women Raises ROS Production by Neural Stem Cells
Source: Dev Neurobiol. 2026 Mar 17;86(2):e70021. doi: 10.1002/dneu.70021 (PMC12996446; doi:10.1002/dneu.70021)
Supplement: Supplementary file 2 — Supplementary Table 1. Statistical results of RT‐qPCR, morphometric, and immunocytochemical analyses performed in BR1 cells and NSCs at passages 2 and 8–9. Supplementary Table 2. Results of cell viability, resazurin reduction, ROS production, and PI staining analyses among the CT, NO, and OB groups at 24, 48, and 72 h. Supplementary Table 3. Results of immunocytochemical and morphometric analyses in NSCs under CT conditions and following treatment with serum from NO and OB individuals. [file DNEU-86-0-s002.docx]

**Supplementary Table 1.** Statistical results of RT-qPCR, morphometric, and immunocytochemical analyses performed in BR1 cells and NSCs at passages 2 and 8–9.

| **CATEGORY** | **OUTCOME** | **BR1**  **(MEAN ± SEM)** | **NSC (P.2)**  **(MEAN ± SEM)** | **NSC (P.8-9)**  **(MEAN ± SEM)** | **STATISTICAL TEST (P)** | **POST-HOC COMPARISONS (P)** |
| --- | --- | --- | --- | --- | --- | --- |
| **mRNA levels** | *NANOG*  (mRNA copy number) | 1182 ± 48.96 | 11.66 ± 1.55 | 59.78 ± 12.34 | One-way ANOVA;  <0.0001 | BR1 vs (p.2); p<0.0001  BR1 vs (p.8-9); p<0.0001  (p.2) vs (p.8-9); p=0.794 |
|  | *OCT4*  (mRNA copy number) | 17.31 ± 3.50 | 17.55 ± 3.13 | 34.10 ± 5.90 | One-way ANOVA;  0.045 | BR1 vs (p.2); p=0.999  BR1 vs (p.8-9); p=0.047  (p.2) vs (p.8-9); p=0.090 |
|  | *SOX2*  (mRNA copy number) | 892.2 ± 9.23 | 1441 ± 74.76 | 887.1 ± 64.72 | One-way ANOVA;  <0.0001 | BR1 vs (p.2); p<0.0001  BR1 vs (p.8-9); p=0.995  (p.2) vs (p.8-9); p<0.0001 |
|  | *FABP7*  (mRNA copy number) | 0.18x10^6^ ± 0.01x10^6^ | 7.77 x10⁶ ± 0.16 x10⁶ | 2.22 x10⁶ ± 0.11 x10⁶ | One-way ANOVA;  <0.0001 | BR1 vs (p.2); p<0.0001  BR1 vs (p.8-9); p<0.0001  (p.2) vs (p.8-9); p<0.0001 |
|  | *NES*  (mRNA copy number) | 59.58 ± 10.20 | 83.56 ± 4.39 | 574.8 ± 39.61 | One-way ANOVA;  <0.0001 | BR1 vs (p.2); p=0.651  BR1 vs (p.8-9); p<0.0001  (p.2) vs (p.8-9); p<0.0001 |
| **Morphometry** | Cell circularity (AU) | 0.58 ± 0.01 | 0.56 ± 0.005 | 0.60 ± 0.007 | One-way ANOVA;  0.066 | BR1 vs (p.2); p=0.541  BR1 vs (p.8-9); p=0.317  (p.2) vs (p.8-9); p=0.056 |
|  | Mean branch length (µm) | 4694 ± 591.5 | 7074 ± 692.1 | 9807 ± 613.8 | One-way ANOVA;  0.005 | BR1 vs (p.2); p=0.049  BR1 vs (p.8-9); p<0.001  (p.2) vs (p.8-9); p=0.036 |
|  | Mean nuclear área (µm^2^) | 3314 ± 154.7 | 3420 ± 55.16 | 3267 ± 147.8 | One-way ANOVA;  0.666 | BR1 vs (p.2); p=0.827  BR1 vs (p.8-9); p=0.963  (p.2) vs (p.8-9); p=0.652 |
| **Immunocytochemistry** | Optical  (Number of cell / 30 μm^2^) | 311.4 ± 10.98 | 297.5 ± 18.62 | 292.7 ± 18.59 | One-way ANOVA;  0.701 | BR1 vs (p.2); p=0.827  BR1 vs (p.8-9); p=0.963  (p.2) vs (p.8-9); p=0.652 |
|  | SOX1 (Integrated density / cell) | 1.328 ± 0.45 | 0.74 ± 0.09 | 150.2 ± 38.35 | One-way ANOVA;  <0.001 | BR1 vs (p.2); p=0.999  BR1 vs (p.8-9); p=0.001  (p.2) vs (p.8-9); p=0.001 |
|  | SOX2 (Integrated density / cell) | 1794 ± 107.9 | 760.4 ± 63.14 | 1170 ± 154.9 | One-way ANOVA;  <0.001 | BR1 vs (p.2); p<0.0001  BR1 vs (p.8-9); p=0.006  (p.2) vs (p.8-9); p=0.065 |
|  | PAX6 (Integrated density / cell) | 435.3 ± 45.60 | 821.5 ± 147.5 | 638.9 ± 99.50 | One-way ANOVA;  0.068 | BR1 vs (p.2); p=0.056  BR1 vs (p.8-9);; p=0.421  (p.2) vs (p.8-9); p=0.494 |
|  | NESTIN (Integrated density / cell) | 6207 ± 1197 | 6322 ± 458.3 | 12112 ± 536.6 | One-way ANOVA;  <0.001 | BR1 vs (p.2); p=0.994  BR1 vs (p.8-9); p<0.001  (p.2) vs (p.8-9); p<0.001 |
|  | FOXG1 (Integrated density / cell) | 0.45 ± 0.06 | 273.4 ± 35.49 | 6.91 ± 2.19 | One-way ANOVA;  <0.0001 | BR1 vs (p.2); p<0.0001  BR1 vs (p.8-9); p=0.959  (p.2) vs (p.8-9); p<0.0001 |

**Supplementary Table 2.** Results of cell viability, resazurin reduction, ROS production, and PI staining analyses among the CT, NO, and OB groups at 24, 48, and 72 hours.

| **ASSAY** | **TIME POINT** | **CT**  **(MEAN ± SEM)** | **NO**  **(MEAN ± SEM)** | **OB**  **(MEAN ± SEM)** | **TWO-WAY ANOVA (Interaction p-value)** | **POST-HOC COMPARISONS (P)** |
| --- | --- | --- | --- | --- | --- | --- |
| **Cell viability**  *% viable cells* | 24h | 97.95 ± 0.29 | 97.65 ± 0.28 | 97.33 ± 0.71 | 0.027 | CT vs NO; p=0.562  CT vs OB; p=0.680  NO vs OB; p=0.909 |
|  | 48h | 97.23 ± 0.21 | 90.83 ± 1.88 | 89.45 ± 1.48 |  | CT vs NO; p=0.042  CT vs OB; p=0.013  NO vs OB; p=0.836 |
|  | 72h | 86.91 ± 1.05 | 75.23 ± 5.01 | 69.49 ± 5.03 |  | CT vs NO; p=0.035  CT vs OB; p=0.052  NO vs OB; p=0.201 |
| **Resazurin**  *Normalized fluorescence*  *(control = 100%)* | 24h | 100 ± 0 | 56.40 ± 1.32 | 59.20 ± 2.39 | 0.003 | CT vs NO; p<0.0001  CT vs OB; p<0.001  NO vs OB; p=0.781 |
|  | 48h | 100 ± 0 | 57.21 ± 2.36 | 68.63 ± 1.52 |  | CT vs NO; p<0.0001  CT vs OB; p<0.001  NO vs OB; p=0.047 |
|  | 72h | 100 ± 0 | 45.76 ± 3.19 | 63.82 ± 2.02 |  | CT vs NO; p<0.001  CT vs OB; p<0.001  NO vs OB; p=0.029 |
| **ROS**  *Normalized fluorescence*  *(control = 100%)* | 24h | 100 ± 0 | 125 ± 2.99 | 127.1 ± 5.34 | 0.002 | CT vs NO; p<0.001  CT vs OB; p=0.008  NO vs OB; p=0.939 |
|  | 48h | 100 ± 0 | 105.8 ± 2.49 | 117.4 ± 1.49 |  | CT vs NO; p=0.142  CT vs OB; p<0.001  NO vs OB; p=0.009 |
|  | 72h | 100 ± 0 | 109.4 ± 3.60 | 128.6 ± 2.25 |  | CT vs NO; p=0.102  CT vs OB; p<0.001  NO vs OB; p=0.004 |
| **Propidium iodide**  *Pixel density (x10^5^)* | 24h | 3.116 ± 0.308 | 4.112 ± 0.195 | 4.336 ± 0.132 | 0.233 | CT vs NO; p=0.204  CT vs OB; p=0.183  NO vs OB; p=0.627 |
|  | 48h | 2.973 ± 0.157 | 4.448 ± 0.104 | 4.910 ± 0.132 |  | CT vs NO; p=0.029  CT vs OB; p=0.008  NO vs OB; p=0.059 |
|  | 72h | 3.358 ± 0.207 | 5.272 ± 0.148 | 5.719 ± 0.449 |  | CT vs NO; p=0.025  CT vs OB; p=0.022  NO vs OB; p=0.489 |

**Supplementary Table 3.** Results of immunocytochemical and morphometric analyses in NSCs under CT conditions and following treatment with serum from NO and OB individuals.

| **CATEGORY** | **OUTCOME** | **CT**  **(MEAN ± SEM)** | **NO**  **(MEAN ± SEM)** | **OB**  **(MEAN ± SEM)** | **STATISTICAL TEST (P)** | **POST-HOC COMPARISONS (P)** |
| --- | --- | --- | --- | --- | --- | --- |
| **Morphometry** | Cell circularity (AU) | 0.65 ± 0.004 | 0.64 ± 0.002 | 0.63 ± 0.003 | One-way ANOVA;  0.009 | CT vs NO; p=0.191  CT vs OB; p=0.009  NO vs OB; p=0.073 |
|  | Mean branch length (µm) | 3247 ± 358.3 | 6858 ± 436 | 6281 ± 170.7 | Kruskal-Wallis;  0.0005 | CT vs NO; p=0.002  CT vs OB; p=0.069  NO vs OB; p=0.891 |
|  | Mean nuclear área (µm^2^) | 2328 ± 115.9 | 3334 ± 238.5 | 3284 ± 245.9 | One-way ANOVA;  0.011 | CT vs NO; p=0.016  CT vs OB; p=0.022  NO vs OB; p=0.985 |
| **Immunocytochemistry** | Optical  (Number of cell / 30 μm^2^) | 732.3 ± 14.80 | 607.7 ± 37.47 | 607.7 ± 28.44 | One-way ANOVA;  P=0.013 | CT vs NO; p=0.023  CT vs OB; p=0.025  NO vs OB; p>0.999 |
|  | SOX1 (Integrated density / cell) | 39407 ± 4298 | 11481 ± 1544 | 9787 ± 2115 | One-way ANOVA;  <0.0001 | CT vs NO; p<0.0001  CT vs OB; p<0.0001  NO vs OB; p=0.891 |
|  | SOX2 (Integrated density / cell) | 25055 ± 2961 | 38679 ± 6243 | 45912 ± 12189 | One-way ANOVA;  0.335 | CT vs NO; p=0.586  CT vs OB; p=0.305  NO vs OB; p=0.824 |
|  | PAX6 (Integrated density / cell) | 10529 ± 984.4 | 381.1 ± 230.7 | 240.2 ± 101.1 | One-way ANOVA;  <0.0001 | CT vs NO; p<0.0001  CT vs OB; p<0.0001  NO vs OB; p=0.969 |
|  | NESTIN (Integrated density / cell) | 85407 ± 6838 | 67272 ± 5203 | 70386 ± 9801 | One-way ANOVA;  0.254 | CT vs NO; p=0.259  CT vs OB; p=0.385  NO vs OB; p=0.952 |
|  | Caspase-3  (Integrated density / cell) | 346.2 ± 87.20 | 737.9 ± 209.6 | 834.4 ± 137.2 | Kruskal-Wallis;  0.037 | CT vs NO; p=0.327  CT vs OB; p=0.040  NO vs OB; p>0.999 |
|  | Ki-67  (Integrated density / cell) | 11020 ± 3240 | 901.5 ± 315.4 | 1198 ± 220.5 | One-way ANOVA;  0.002 | CT vs NO; p=0.003  CT vs OB; p=0.005  NO vs OB; p=0.992 |
|  | p53  (Integrated density / cell) | 268.4 ± 63.14 | 1486 ± 144.7 | 2126 ± 338.1 | One-way ANOVA;  0.0001 | CT vs NO; p=0.002  CT vs OB; p=0.001  NO vs OB; p=0.107 |
|  | NeuN  (Integrated density / cell) | 162.2 ± 25.68 | 2301 ± 469.8 | 2000 ± 322.5 | Kruskal-Wallis;  0.002 | CT vs NO; p=0.016  CT vs OB; p=0.023  NO vs OB; p>0.999 |
